# Disseminating Trauma-Focused Cognitive Behavioral Therapy with a Systematic Self-care Approach to Addressing Secondary Traumatic Stress: PRACTICE What You Preach

**DOI:** 10.1007/s10597-020-00602-x

**Published:** 2020-04-21

**Authors:** Esther Deblinger, Elisabeth Pollio, Beth Cooper, Robert A. Steer

**Affiliations:** 1 CARES Institute, Rowan University School of Osteopathic Medicine (RowanSOM), 42 E. Laurel Road, Stratford, NJ 08084 USA; 2 Department of Psychiatry, Rowan University School of Osteopathic Medicine (RowanSOM), 42 E. Laurel Road, Stratford, NJ 08084 USA

**Keywords:** TF-CBT, Childhood trauma, Dissemination, Secondary traumatic stress, Self-care

## Abstract

This pilot study evaluated the effectiveness of Trauma-Focused Cognitive Behavioral Therapy (TF-CBT) training programs augmented with a systematic “PRACTICE What You Preach” (PWYP) self-care focus, which has trainees personally utilize the coping skills they teach their clients. Participants were 115 clinicians/supervisors who completed a PWYP TF-CBT training program. Pre- to post-training analyses documented significant increases in participants’ competency and fidelity in implementing TF-CBT (*p*s < .001), significantly more frequent use of coping skills including instrumental social support (*p* < .01), active coping (*p* < .001), humor (*p* < .01), and restraint (*p* < .01), and significant decreases in secondary traumatic stress (STS; *p* < .001). Children’s symptoms of PTSD (*p*s < .001) and behavior problems (*p* < .05) also decreased significantly. This preliminary evidence suggests that training augmented with PWYP may enhance clinicians’/supervisors’ personal coping and reduce their levels of STS without compromising treatment implementation efforts and client outcomes.

## Introduction

Childhood traumas may underlie the emotional and behavioral problems that many children experience (e.g., Dye 2018; Moylan et al. 2010). In fact, childhood traumas, such as sexual abuse, exposure to domestic or community violence, and traumatic losses have been found to negatively impact the adjustment and development of a significant proportion of youth (Saunders and Adams 2014). More specifically, study findings have repeatedly suggested links between childhood trauma and the development of posttraumatic stress disorder (PTSD), clinical depression, suicidal behavior, substance abuse, and physical health problems that develop in childhood and may last a lifetime (e.g., Felitti et al. 1998; Thompson et al. 2015). Given the severe and disruptive effects of trauma, it is critical that children across all social, economic, ethnic, and racial groups have equal access to effective mental health interventions to address trauma-related stress reactions.

Over the last several decades, efforts have been made to develop psychosocial interventions to forestall this myriad of negative traumatic stress effects on youth. One such evidence-based intervention, Trauma-Focused Cognitive Behavioral Therapy (TF-CBT; Cohen et al. 2017; Deblinger et al. 2015), has been reported to be one of the most well-researched and widely disseminated of the available trauma-informed, evidence-based practices (Allen and Johnson 2012). TF-CBT, designed for children ages 3 to 18 years and their non-offending caregivers, has demonstrated sustained benefits in numerous randomized trials and can be delivered in individual or group format (e.g., Cohen et al. 2004; Deblinger et al. 2001, 2006, 2016). The acronym PRACTICE reflects the components of treatment, which include **P**sychoeducation, **P**arenting skills, **R**elaxation skills, **A**ffective expression and modulation skills, **C**ognitive coping skills, **T**rauma narration and processing, **I**n vivo mastery, **C**onjoint child-parent sessions, and **E**nhancing safety and development. This model has been evaluated and documented to be highly effective in addressing trauma-related difficulties in over 50 scientific investigations including at least 20 randomized controlled trials (Cohen et al. 2017; Deblinger et al. 2015; Mannarino et al. 2014). Research has also documented the cost-effectiveness of TF-CBT as compared to other treatment approaches (Aas et al. 2018; Greer et al. 2014). Although this treatment model is considered the standard of care for youth impacted by abuse, violence, and other childhood traumas (Leenarts et al. 2013), many families seen at community mental health agencies have been unable to access this evidence-based trauma-focused intervention. As a result, there have been increasing efforts to train clinicians in TF-CBT through a combination of online introductory training and statewide in-person training initiatives (Cohen and Mannarino 2008; Heck et al. 2015; Sigel et al. 2013a, b).

Training and dissemination studies have documented that high rates of staff turnover can undermine the efforts of community mental health agencies to train their staff in effective evidence-based practices and sustain those practices after the training period (Aarons et al. 2011; Swain et al. 2010; Woltmann et al. 2008). Research also suggests that over a 1-year period, turnover rates for mental health staff at community agencies are as high as 50% (Woltmann et al. 2008). One variable shown to contribute to staff turnover is a high rate of secondary traumatic stress (STS) among clinicians (Salloum et al. 2015). STS has been described as experiencing emotional distress or impairment resulting from hearing about the experiences of trauma survivors (Bride 2007; NCTSN 2011). Moreover, research has documented higher rates of STS and burnout among mental health clinicians who work in the field of trauma (Bride 2007; Cieslak et al. 2014; Hamama 2012). Those with a higher proportion of traumatized clients or more time spent with trauma survivors may be at greater risk for experiencing these difficulties (Hensel et al. 2015). Although numerous empirical studies have documented the nature, prevalence, and impact of the stressors experienced by mental health clinicians working with trauma survivors (Bride 2007; Creamer and Liddle 2005; Cunningham 2003; Hamama 2012; Ireland and Huxley 2018), a systematic review of the empirical literature found minimal research on interventions designed to prevent and/or ameliorate the stressful effects of this work on the clinicians delivering therapy to this vulnerable population (Bercier and Maynard 2015).

The purpose of the present study was to conduct a preliminary evaluation of the impact of systematically encouraging and following up on training participants’ personal use of coping skills taught during the TF-CBT training sessions to reduce daily stress and job-related emotional strain. More specifically, the coping skills encouraged were the very ones clinicians/supervisors were taught to encourage their clients to use in the course of implementing TF-CBT. This self-care emphasis, referred to as “PRACTICE What You Preach” (PWYP), was designed to be integrated into standard TF-CBT training to help reduce possible STS in clinicians/supervisors who work with trauma survivors. In addition to potentially reducing trainees’ stress levels, this self-care approach was designed to augment the positive effects of TF-CBT training on self-reported TF-CBT competency, treatment fidelity, and client outcomes that have been reported in the literature to date (Amaya-Jackson et al. 2018; Sigel et al. 2013a, b; Woody et al. 2015).

Although the present study is not a randomized trial, it examines preliminary data on clinicians’/supervisors’ coping strategies and levels of STS before and after participation in the PWYP TF-CBT training program. More specifically, it was hypothesized that clinicians/supervisors who participated in the PWYP TF-CBT training program would demonstrate increases in the personal use of effective coping skills and decreased levels of STS. In addition, the clinicians/supervisors were expected to report enhanced TF-CBT competency and fidelity. Finally, it was anticipated that, consistent with prior research, youth treated with TF-CBT would show significant decreases in PTSD and behaviors problems.

## Methods

Research has documented that one-time only workshops do little to produce change in professional practices (e.g., Ebert et al. 2008; Lyon et al. 2011). Thus, the current study recruited clinicians from mental health agencies to participate in an intensive TF-CBT training program, with multiple learning sessions and consultation, augmented with the PWYP approach. Agencies were encouraged to apply for training if (1) they provided services to youth and caregivers impacted by abuse, violence, or other traumas, (2) they had the capacity to provide therapy to parents/caregivers separate from and in conjunction with the children (e.g., oversight of children in waiting area), thus allowing caregivers to be seen by therapists to learn to manage their own stress while providing support to their youngsters, (3) they would utilize standardized measures to objectively assess the mental health adjustment of clients pre- and post-treatment, and (4) they had clinicians and supervisors who were interested in implementing TF-CBT with appropriate clients.

Prior to participating in the training program, 168 trainees were asked to provide research consent for their survey responses. Participants were informed that they would receive the same training regardless of whether or not they provided research consent. Ninety-six percent (*N* = 161) agreed to participate in the research. Only data from the trainees who provided informed consent were included in the present study. This study was approved by the Institutional Review Board at Rowan University School of Osteopathic Medicine.

### Participants

There were 115 mental health clinicians/supervisors from five training cohorts who successfully completed the TF-CBT training program that utilized the PWYP approach between November 2014 and August 2017. Successful completion entailed attending all days of in-person training, actively participating on at least 80% of the 18 consultation calls where TF-CBT training cases were presented, completion of pre- and post-training surveys, and the successful completion of at least one TF-CBT training case. Successful completion of a training case is defined as the completion of the TF-CBT PRACTICE components with an appropriate TF-CBT case and the submission of at least one standardized assessment measure at pre- and post-treatment. Of note, agency senior leaders were also involved in the training program to discuss organizational issues related to TF-CBT implementation, but their data were not collected unless they were also participating in a supervisory role and directly providing services to clients. Participants were from 19 agencies predominantly across New Jersey, with one agency in Brooklyn, New York, and another agency with locations across Florida. Two participants were private practitioners. The demographic characteristics of the 161 trainees who agreed to participate in research are shown in Table 1.

**Table 1:** Comparisons of the demographic characteristics and pre-training scores of the clinicians who did and did not complete training

Variable	Completers	Non-completers	χ^2^_Yates_ (N = 161)	Φ
	(N = 115)	(N = 46)		
% Female	90	80	1.68	.12
% Caucasian	88	74	3.73	.17
% MSW	55	41	1.88	.12
% Licensed	82	70	2.19	.13
% Employed > 2 years at current agency	50	37	1.63	.11
% CBT oriented	57	56	0.01	.01
% Supervisors	28	28	0.00	.00

	Completers		Non-completers		*t/t'*	*(dfs)*	*d*
	(N = 115)		(N = 46)				
	M	SD	M	SD			
Age (years)	37.25	9.51	39.98	12.41	1.34	67	.26
ProQOL-5 subscales							
Compassion satisfaction	41.90	4.43	41.91	5.25	0.01	159	.00
Burnout	19.53	4.13	20.35	5.07	0.97	70	.19
Secondary traumatic stress	19.20	4.29	19.61	4.66	0.53	159	.09
COPE subscales							
Positive reinterpretation and growth	13.52	2.15	13.41	2.27	0.29	159	.05
Mental disengagement	8.55	2.07	8.54	2.20	0.01	159	.00
Venting of emotions	9.90	2.46	9.78	2.64	0.26	159	.04
Instrumental social support	12.30	2.32	12.07	2.54	0.55	159	.10
Active coping	12.39	1.93	12.39	2.02	0.00	159	.00
Humor	10.15	3.02	10.04	3.56	0.19	159	.03
Behavioral disengagement	5.51	1.84	5.50	1.49	0.04	159	.01
Restraint	10.58	2.18	10.72	2.00	0.36	159	.06
Emotional social support	12.89	2.63	12.22	2.98	1.40	159	.24
Suppression of competing activities	10.04	2.09	10.33	2.14	0.77	159	.13
Planning	13.51	1.87	13.39	2.28	0.35	159	.06
Acceptance	11.85	1.98	11.04	2.47	2.18	159	.38

	Completers		Non-completers		*t/t'*	*(dfs)*	*d*
	(N = 82)		(N = 36)				
Fidelity Checklist Total Scores	168.62	34.53	162.39	31.02	0.93	116	.17
Competency Survey Total Scores	59.72	14.81	54.33	17.56	1.72	116	.32

*MSW* masters of social work, *CBT* cognitive behavioral therapy, *ProQOL-5* professional quality of life scale version 5. *Fidelity Checklist* TF-CBT PRACTICE Fidelity Checklist, *Competency Survey* TF-CBT Competency Self-Report Survey; t values with different degrees of freedom are for Welch's t'

MANOVA used to compare the completers and non-completers with respect to the set of 16 coping and stress scales in the ProQOL-5, COPE, and PSS was not significant, MANOVA F(16, 144) = 0.66, p = .83, Hotelling Trace = .07

### Procedures

**Fig. 1:**
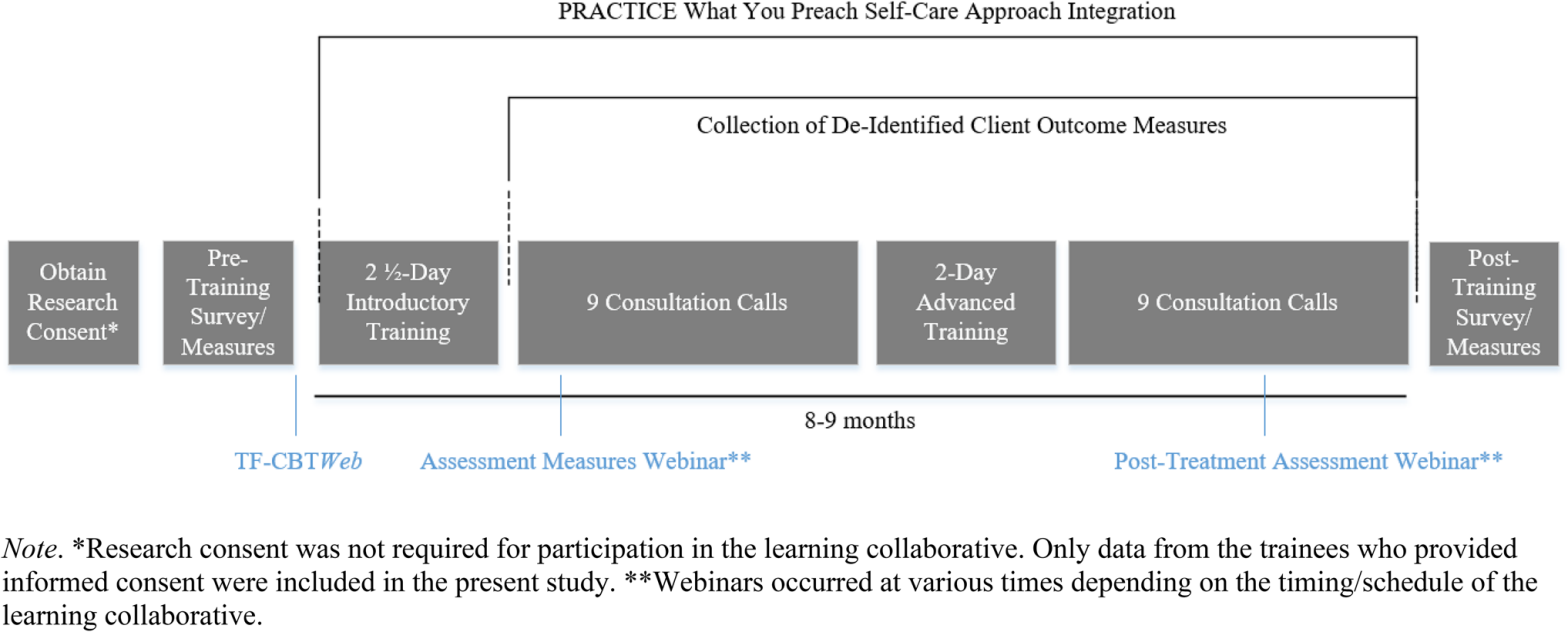
TF-CBT PWYP Learning Collaborative Timeline

Over the course of approximately 8 to 9 months, participants received extensive training in TF-CBT that systematically focused on self-care from the start. The training program utilized a modified version of learning collaborative methodology (Ebert et al. 2008) that consisted of an online TF-CBT overview (now available at https://tfcbt2.musc.edu), a 2½-day in-person introductory training, assessment measures webinars, a 2-day advanced training, and 18 consultation calls with an expert in TF-CBT. Half of the consultation calls occurred following the introductory training but prior to the advanced training, and the other half occurred after the advanced training. Figure 1 displays the modified learning collaborative methodology used for the training programs. Consultation with experts, also referred to as expert coaching, has demonstrated effectiveness with helping clinicians learn a model and utilize the model with greater fidelity (e.g., Lyon et al. 2011).

The self-care focus began in the face-to-face introductory training with educational presentations highlighting the potential benefits of practicing the skills clinicians/supervisors promote to clients. Examples and exercises highlighting the personal use of evidence-based coping skills (PWYP skills) were integrated into all of the introductory and advanced training days in a standardized way to promote consistency across the five training cohorts. Participants were engaged in exercises designed to reduce their daily stress and enhance their appreciation for the impact the coping skills had on their personal well-being and interactions. It was also anticipated that practicing the skills might increase the clinicians’/supervisors’ abilities to inspire clients to implement those same coping skills between sessions. Examples of skills participants practiced included using specific praise with someone in their personal lives, completing a thought record to challenge an unhelpful or inaccurate thought, and utilizing focused breathing when faced with a stressful situation. Participants were provided simple items that served as reminders for them to practice their coping skills on a daily basis (e.g., water bottles and magnets with the PWYP logo).

Each consultation call had an agenda that utilized the following basic structure: attendance, review of homework (referred to as PRACTICE assignments) including the use of PWYP skills and a clinical assignment (e.g., read a chapter from the treatment manual), case presentations and discussion, topics to highlight during the case presentation, and description of the PRACTICE assignments for the next call. Across the 18 calls, approximately four hours of the consultation call time (i.e., 10–20 min per call) were specifically designed to focus on the participants’ use of the PWYP skills in their daily lives (professional and personal). As previously noted, consultation calls started with a review of the participants’ use of PWYP skills, which parallels the process of clinicians reviewing PRACTICE assignments with clients at the beginning of a TF-CBT session. The consultants asked the participants to describe their use of the skill, including challenges and successes, and then provided feedback to the participants. Participants were held accountable for completing their PRACTICE assignment, as clinicians would do with TF-CBT clients, and the consultants modeled for the trainees the process of reviewing the PRACTICE assignments with their clients by doing the same with the PWYP assignment at the beginning of each call.

The trainees’ levels of burnout, compassion satisfaction, STS, and use of coping skills, as well as fidelity and competency with respect to the implementation of TF-CBT, were measured pre- and post-training. Clinicians/supervisors were also required to submit de-identified pre- and post-treatment outcome measures of PTSD symptoms and behavioral difficulties exhibited by clients.

### Trainee Survey Measures

The surveys were administered through online data collection tools (Qualtrics or Survey Monkey) at pre- and post-training. If requested by a trainee, paper surveys were provided and responses from paper surveys were entered into an online data collection tool by the research assistant for the project. The surveys included questions to gather demographic information as well as measures to assess trainees’ levels of burnout, STS, use of coping skills, and self-reported fidelity and competency related to TF-CBT implementation. Additionally, participants were asked to rate how frequently they utilized PWYP skills, their reactions to the focus on these skills, and the benefits of practicing them in their personal and professional lives.

#### Professional Quality of Life Scale

The Professional Quality of Life Scale 5 (ProQOL-5; Stamm 2009) is a 30-item self-report measure that assesses a professional’s compassion satisfaction, burnout, and STS. Respondents are asked to use a 5-point rating scale ranging from *never* to *very often* to describe how frequently in the past 30 days they experienced certain thoughts and feelings related to their work as a “helper.” Items load onto three subscales: compassion satisfaction, burnout, and STS. The ProQOL-5 has been found to have adequate internal consistencies. Hemsworth et al. (2018) reported Cronbach coefficient αs ≥ .70, and Stamm (2010) described coefficient αs ranging from .75 to .88. The ProQOL-5 has also demonstrated construct validity with respect to other measures (Stamm 2010).

#### COPE

The COPE (Carver et al. 1989) is a 60-item self-report measure that assesses a broad range of coping responses to difficult or stressful events. There are 15 subscales, each of which identifies specific coping mechanisms. For the purposes of the present study, 12 of the subscales were utilized. Respondents are asked to indicate how often they utilize a particular coping response when experiencing stressful events. The responses are rated on a 4-point scale ranging from *I usually don’t do this at all* to *I usually do this a lot*. Higher scores indicate more frequent use of that coping response. The COPE has adequate internal reliability for research purposes with a median Cronbach coefficient α across its subscales of .73 (Litman 2006). Carver et al. (1989) reported that there was only one subscale with a coefficient α < .60 (mental disengagement). With respect to its construct validity, the self-sufficient and socially-supported coping subscales were correlated with behavioral activation, positive traits, and approach-oriented coping, whereas the avoidant-coping subscales were correlated with behavioral inhibition and negative traits (Litman 2006).

#### TF-CBT PRACTICE Fidelity Checklist

The TF-CBT PRACTICE Fidelity Checklist (Fidelity Checklist; Deblinger et al. 2014) is a 48-item self-report measure designed to assess fidelity to the components of TF-CBT. Respondents are asked to use a 5-point rating scale ranging from *never* to *almost always* to describe how often they have used various treatment strategies with their TF-CBT clients during the past four months. The Fidelity Checklist was derived from the *PRACTICE Checklist Self-Report* developed by Deblinger et al. (2005). In studies conducted by Ebert et al. (2012) and Hanson et al. (2018), the Cronbach coefficient αs for the *PRACTICE Checklist Self-Report* were > .95. In the present study, the Cronbach coefficient αs for the Fidelity Checklist pretest and posttest total scores were, respectively, .97 and .96.

#### TF-CBT Competency Self-report Survey

The TF-CBT Competency Self-report Survey (Competency Survey; CARES Institute 2014) is a 20-item measure of clinicians’ self-reported level of competence in implementing TF-CBT skills and related activities. Respondents are asked to rate on a 5-point scale ranging from *not at all* to *exceptionally* how competent they feel in implementing TF-CBT-related skills and activities including using the PRACTICE components, identifying appropriate cases, and balancing flexibility with fidelity, as well as adapting the model to the needs of different clients and trauma types. The Competency Survey was derived from the *TF-CBT Clinical Skills Questionnaire* developed by Saunders and colleagues (National Crime Victims Research and Treatment Center, Medical University of South Carolina 2010). In a study conducted by Dopp et al. (2017), the Cronbach coefficient αs for the *TF-CBT Clinical Skills Questionnaire* was > .98. In the present study, the Cronbach coefficient αs for the Competency Survey pretest and posttest total scores were, respectively, .97 and .95.

#### PRACTICE What You Preach Behaviors and Activities Questions

The PRACTICE What You Preach Behaviors and Activities questions (PWYP questions) were developed for this study and reflected the nine self-care behaviors and activities encouraged over the course of the training program. Clinicians/supervisors were asked to use a 5-point rating scale ranging from *never* to *very often* to describe how frequently they engaged in the PWYP behaviors and activities during the previous four months. Table 2 provides short descriptions for these nine PWYP questions.

**Table 2:** List of short descriptions for PRACTICE what you preach behaviors and activities questions

Shortened label	Item
Physical activity	I engaged in physical activities (e.g., walking, cycling, dancing, yoga, running, exercise, etc.)
Social activities	I engaged in enjoyable social activities
Mindfulness	I focused on the present moment (e.g., mindfulness, meditation, etc.) as opposed to the past or the future
Gratitude/praise	I expressed gratitude and/or acknowledged others with praise for specific things they have done (i.e., more than a simple thank you)
Reflective listening	In my personal life, I validated others’ feelings by listening without giving advice
Focused breathing	I took a few deep breaths to help me relax
Cognitive coping	I noticed when my thoughts were too negative and I made a conscious effort to think in more optimistic ways
Writing activity	I wrote down my deepest thoughts and feelings about a situation
PRACTICE skills	I practiced the skills I encouraged my clients to practice

### Client Outcome Measures

#### Child Behavior Checklist

The Child Behavior Checklist (CBCL; Achenbach and Rescorla 2001) is one of the most widely used caregiver-report measures of children’s/adolescent’s behavioral and emotional problems as well as social competence. There are two versions: one for caregivers of children ages 1.5 to 5 years, and another for caregivers of children ages 6 to 18 years. Caregivers are asked to rate how true each of the 120 items is for their child using a 3-point scale ranging from *not true* to *very true or often true*. The scale has good reliability and validity (Nakamura et al. 2009). Coefficient αs ranging from .72 to .97 have been reported (Achenbach et al. 2008; Achenbach and Rescorla 2001; Jastrowski Mano et al. 2009). For this study, the Externalizing and Internalizing subscales, which have been found to display good internal consistency and criterion-related validity (Achenbach et al. 2008), were used to measure pre- to post-treatment changes.

### PTSD Measures

#### UCLA Posttraumatic Stress Disorder Reaction Index for DSM-IV

The UCLA Posttraumatic Stress Disorder Reaction Index for DSM-IV (UCLA PTSD-RI for DSM-IV; Steinberg et al. 2004) is a brief measure that assesses exposure to traumatic events and associated DSM-IV PTSD symptoms in children and adolescents. There are three versions of the measure: a self-report version for children; a self-report version for adolescents; and a caregiver-report version. Symptom items are scored on a 5-point scale ranging from *none* to *most*. Reponses load onto Criterion B (reexperiencing), Criterion C (avoidance), and Criterion D (increased arousal) for PTSD. Total scores are derived by adding criterion B, C, and D subscale scores (Steinberg et al. 2013). This measure has demonstrated good to excellent internal consistency reliability with Cronbach coefficient αs ranging from .85 to .91 (Steinberg et al. 2004, 2013).

#### UCLA Posttraumatic Stress Disorder Reaction Index for DSM-5

The UCLA PTSD-RI for DSM-IV was updated to correspond to the diagnostic criteria in the DSM-5. There are two versions of the UCLA PTSD-RI for DSM-5 (Pynoos and Steinberg 2015): a self-report version for children and adolescents ages 7 to 18 years, and a caregiver-report version. Symptom items are scored on a 5-point scale ranging from *none of the time* to *most of the time*. Reponses load onto Criterion B (intrusion), Criterion C (avoidance), Criterion D (negative cognitions/mood), and Criterion E (arousal/reactivity) for PTSD. Total scores are derived by adding criterion B, C, D, and E subscale scores. Recent research on the psychometric properties supports excellent internal consistency, criterion-referenced validity, and diagnostic accuracy through discriminating children diagnosed with PTSD from other trauma-exposed children who did not meet criteria for PTSD (Kaplow et al. 2020; Rolon-Arroyo et al. 2017). Cronbach α scores ranged from .76 to .89, with only Criterion C falling below this range. The internal consistency of the total score was excellent, with an α score of .94 (Kaplow et al. 2020).

Two different versions of the UCLA PTSD-RI were used by the training cohorts with the latter cohorts using the more up-to-date DSM-5 version when it became available. Statistically, for the purposes of this study, utilizing two versions did not impact the results because each child’s and caregiver’s pre-and post-treatment PTSD scores were compared using paired *t* tests for the same instrument (e.g., if the UCLA PTSD-RI for DSM-IV was completed at pre-treatment by a particular client, the UCLA PTSD-RI for DSM-IV was completed at post-treatment by that client). Therefore, further references to the UCLA PTSD-RI are inclusive of both versions.

## Results

Although 161 trainees participated in the training program and provided consent for research, 46 (29%) trainees did not successfully complete the program. The most common reasons for not completing the training program were not successfully completing a TF-CBT training case (54%), leaving their agency (17%), and not getting a training case (7%). Six trainees (13% of non-completers) discontinued their participation in the training program without providing a reason.

Due to concerns that the trainees who completed the training program might reflect a biased sample, we decided to determine whether the pre-training background characteristics and stress levels of the trainees who did complete the training program (completers; *N* = 115, 71%) were comparable to the 46 (29%) who did not complete the training program (non-completers). Table 1 shows the comparisons of the participants’ pre-training demographic characteristics, stress levels, and Fidelity Checklist and Competency Survey scores by training program completion status. To control for the familywise error rates of comparing the 24 variables listed in Table 1, a Bonferroni adjustment of .05/8 (*p* > .00625) for alpha was used to establish the significance level of alpha, two-tailed test, for the seven demographic categorical characteristics along with age. Multivariate analyses of variance (MANOVAs) were used to establish the ceiling levels for alpha with respect to the ProQOL-5 and COPE as well as the Fidelity Checklist and Competency Survey. None of the *χ*^2^ tests for independence with a Yates correction for continuity or the *t* tests for independence listed in Table 1 was significant. Furthermore, the mean set of scores for the ProQOL-5 and COPE for the completers and non-completers were also comparable, MANOVA *F* (15, 145) = .71, *p* = .77, Wilks’ *λ* = .93. Likewise, the mean set of scores for the Fidelity Checklist and Competency Survey scores did not significantly differ, MANOVA *F* (2, 115) = 1.53, *p* = .22, Wilks’ *λ* = .97. Therefore, none of the *t* tests for independence shown in Table 1 were significant. The effect sizes calculated as *φ*s for the categorical demographic characteristics and as *d* statistics for the continuous variables are listed in Table 1 to indicate that the mean differences between the completers and non-completers across the scales were small according to Cohen’s (1992) interpretative guidelines. Therefore, we concluded that 115 clinicians who completed the training program were similar to the 46 clinicians who did not complete the program.

Repeated MANOVAs were next performed to establish the ceiling for alpha with respect to the set of pre- and post-training mean differences for subscales measured by the ProQOL-5 and the COPE, the set of nine self-care behaviors encouraged during the training program (PWYP questions), and the set of two Fidelity Checklist and Competency Survey total scores. The set of mean changes over time for the ProQOL-5 and COPE subscales (MANOVA *F*(15, 99) = 3.00, *p* < .001, Wilks’ *λ* = .670), the PWYP questions (MANOVA *F*(9, 106) = 2.62, *p* < .01, Wilks’ λ = .82), and the Fidelity Checklist and Competency Survey Scales (MANOVA *F*(2, 80) = 2.59, *p* < .001, Wilks’ λ = .82) were significant. Therefore, Table 3 displays the pre- and post-training means, standard deviations, Pearson correlations, paired *t* tests, and *d* statistics for the ProQOL-5 and COPE, the ratings for the PWYP questions, as well as the Fidelity Checklist and Competency Survey total scores. The effect sizes for the paired *t* tests were calculated according to procedures recommended by Dunlap et al. (1996). As Table 3 indicates, the completers’ mean level of STS as measured by the ProQOL-5 significantly decreased, and the use of instrumental social support, active coping, humor, and restraint as measured by the COPE significantly increased. With respect to the PWYP questions, the use of the following self-care skills increased significantly: engaging in physical activity, taking deep breaths as a relaxation strategy, writing down thoughts and feelings, and generally personally practicing skills taught to clients. The aforementioned mean changes were significant and represented small effects sizes. The post-training Fidelity Checklist and Competency Survey mean total scores were also significantly higher at post-training. The mean difference in the Fidelity Checklist scores represented a large effect size of .83, whereas the mean increase in the Competency Survey scores reflected a medium effect size.

**Table 3:** Comparison of pre- and post-training mean stress scores, behaviors, and activities for clinicians who completed training

Variable	Pre-training (N = 115)		Post-training (N = 115)		*r*	*t(114)*	*d*
	M	SD	M	SD			
ProQOL-5 subscales							
Compassion satisfaction	41.90	4.43	42.09	4.78	.65	0.51	.04
Burnout	19.53	4.13	19.43	4.06	.67	0.31	.02
Secondary traumatic stress	19.20	4.29	17.74	4.45	.59	3.98***	.34
COPE subscales							
Positive reinterpretation and growth	13.52	2.15	13.75	1.81	.56	1.30	.11
Mental disengagement	8.55	2.07	8.70	1.98	.57	0.89	.08
Venting of emotions	9.90	2.46	10.05	2.23	.52	0.73	.07
Instrumental social support	12.30	2.32	12.89	2.29	.63	3.20**	.26
Active coping	12.39	1.93	12.98	1.81	.50	3.38***	.32
Humor	10.15	3.02	10.96	3.28	.63	3.18**	.26
Behavioral disengagement	5.51	1.84	5.76	1.71	.30	1.24	.14
Restraint	10.58	2.18	11.13	2.04	.52	2.86**	.26
Emotional social support	12.89	2.63	12.95	2.56	.70	0.33	.02
Suppression of competing activities	10.04	2.09	10.40	2.19	.31	1.52	.17
Planning	13.51	1.87	13.66	1.91	.42	0.78	.08
Acceptance	11.85	1.98	11.87	2.32	.48	0.08	.01
PWYP questions							
Physical activity	3.80	1.08	4.01	0.94	.65	2.62*	.20
Social activities	3.97	0.78	4.03	0.78	.55	0.76	.07
Mindfulness	3.69	0.78	3.78	0.79	.37	1.19	.12

	Pre-training (N = 115)		Post-training (N = 115)		*r*	*t(114)*	*d*
	M	SD	M	SD			
Gratitude/praise	4.22	0.75	4.13	0.69	.45	1.23	.12
Reflective listening	3.99	0.68	3.97	0.64	.22	0.23	.03
Focused breathing	4.07	0.76	4.29	0.75	.14	2.36*	.29
Cognitive coping	3.82	0.76	3.97	0.74	.37	1.89	.20
Writing activity	2.10	1.05	2.41	1.25	.43	2.62*	.26
PRACTICE skills	3.85	0.67	4.02	0.66	.34	2.33*	.25

	Pre-training (N = 82)		Post-training (N = 82)		*r*	*t(81)*	*d*
	M	SD	M	SD			
Fidelity Checklist Total Scores	168.62	34.53	199.24	25.75	.42	8.30 ***	.83
Competency Survey Total Scores	59.72	14.81	81.65	9.54	.42	14.34 ***	.44

*ProQOL-5* professional quality of life scale version 5, *PWYP* PRACTICE what you preach behaviors and activities questions, *Fidelity Checklist* TF-CBT PRACTICE fidelity checklist, *Competency Survey* TF-CBT competency self-report survey

*p < .05, **p < .01, ***p < .001

Over the course of the training programs, 115 trainees (which included both clinicians and supervisors implementing TF-CBT with youth) participated in the pre-training webinars and activities, completed pre-post surveys, attended the two face-to-face trainings, participated in at least 80% of the consultation calls, and completed treatment including all of the relevant PRACTICE components with at least one child/adolescent. Among the trainees, 110 of the participants submitted at least one pre- and post-treatment standardized measure (i.e., the CBCL and UCLA PTSD-RI measures) for a successfully completed treatment case. However, five trainees, who completed all other training requirements, did not have a pre-post assessment measure for a successful training case included in the analyses. There were a number of reasons that this occurred. In one case, the caregiver changed over the course of treatment and the child was too young to complete measures; although a post-treatment assessment measure was completed by the new caregiver, no pre-post analyses were able to be conducted for this case. In another case, the clinician submitted post-treatment assessment measures for a training case but the measures were illegible and unable to be scored, thus they were not included in the data set. For the remaining three clinicians, the requirement for the submission of at least one standardized assessment measure at post-treatment was waived because although their clients completed a full course of TF-CBT as confirmed by the consultant, the clients did not attend the post-treatment assessment appointment for reasons out of the clinician’s control.

It should also be noted that although clinicians were encouraged to complete both the UCLA PTSD-RI and the CBCL, there were a number of successful treatment cases in which only one post-treatment measure was completed. The following are examples of reasons that only one measure was completed for certain cases: there was no participating caregiver; the caregiver dropped out of treatment, as such there was no post-treatment assessment measure submitted for the caregiver; the caregiver changed over the course of treatment so no pre-post comparison was able to made; the child was too young to complete measures; only one caregiver report measure was submitted (i.e., either the CBCL or UCLA PTSD-RI).

Table 4 shows the mean pre- and post-training CBCL and UCLA PTSD-RI scores for the children and caregivers who had completed at least one set of pre- and post-training CBCL or UCLA PTSD-RI measures. A Bonferroni adjustment for alpha of .05/5 (.01) was used to control for the familywise error rate of performing 5 paired *t* tests. Table 4 lists the pre- and post-training means, standard deviations, Pearson correlations, paired *t* tests, and *d* statistics for the CBCL Internalizing, Externalizing, and total scores. This included 93 caregivers for whom complete pre- and post-treatment CBCL data as *T*-scores were available along with the UCLA PTSD-RI mean scores as reported by 95 youth themselves and by 93 of their caregivers. The pre-and post-training differences for the means displayed in Table 4 represented highly significant improvements (*p*s < .001). The *d* statistics for the pre-and post-training mean difference in the CBCL externalizing scores represented a medium effect size, and the magnitudes of the *d* statistics for both the CBCL internalizing and UCLA PTSD-RI youth-report and caregiver-report total symptom scores reflected large effect sizes.

**Table 4:** Comparison of children's mean pre- and post-treatment CBCL and UCLA PTSD-RI

Scale	N	Pre-treatment		Post-treatment		*r*	*t*	*df*	*d*
		M	SD	M	SD				
CBCL									
Internalizing	93	62.43	10.13	54.71	10.92	.61	7.96***	92	.83
Externalizing	93	59.97	10.65	54.83	11.64	.67	5.47***	92	.56
Total Scores	93	62.53	9.53	55.14	10.46	.71	9.34***	92	.96
UCLA PTSD-RI									
Youth report	95	34.08	16.95	20.01	13.48	.52	9.00***	94	.92
Parent/caregiver report	93	30.83	13.35	17.56	11.25	.56	10.76***	92	1.12

*CBCL* child behavior checklist, *T*-scores, *UCLA PTSD-RI* UCLA Posttraumatic Stress Disorder Reaction Index

***Bonferroni adjusted, alpha/5: p < .001

Through their survey responses, the trainees also provided qualitative data sharing the benefits of focusing on self-care and personally utilizing the skills they were teaching their clients. When asked about their reaction to the focus on PWYP skills, responses included: “‘Yes!!! Please talk about this!!!’ That was my reaction. I believe we don’t talk about self-care enough in the mental health field, especially when providing trauma focused treatment;” “I thought that it was good… and necessary in this line of work. Talking about it and then actually making sure that we did it, are two different things.” When asked about the benefits of practicing PWYP skills in their personal and professional lives, responses included: “When using the skills, I feel more relaxed and better able to cope with my daily life stressors;” “Less stress, more appreciation for my work setting, generally more fun to be at work;” “I have a different perspective on what I ask my clients to do and realizing how hard some of these things are has been an eye opening experience. I think that this has changed me in the way that I do treatment in understanding how hard it is and to be more supportive.”

## Discussion

The present findings of this investigation provide preliminary support for the potential benefits of incorporating a PWYP self-care approach into TF-CBT training. The results demonstrated some increases in the use of effective coping skills among trainees, and there were significant decreases in their levels of STS. There were also significant increases in the trainees’ reported treatment fidelity, and their beliefs about their competency and ability to implement TF-CBT. During this 8 to 9-month PWYP TF-CBT training program, significant improvements on child outcomes with respect to PTSD and behavior problems were also observed. Consequently, the systematic augmentation of standard TF-CBT training with an approach emphasizing self-care does not seem to have diminished the trainees’ fidelity and beliefs that they can competently implement TF-CBT, nor does such a focus seem to diminish the expected client symptom reductions.

The positive changes in feelings of competence are critically important because TF-CBT requires clinicians to engage in activities with children and their caregivers that are challenging, and it is essential for therapists to have confidence in the benefits of the model and their skills in implementing it. The participants’ post-training increases in reported levels of fidelity to the TF-CBT model are also critically important because previous research has found that greater treatment fidelity was associated with better treatment outcomes for children who have experienced trauma (Amaya-Jackson et al. 2018).

Furthermore, the present study demonstrated a higher participant completion rate at 71% as compared to other known completion rates for standard TF-CBT training programs. For example, Sigel et al. (2013b) reported difficulties in gaining buy-in from their mental health trainees and reported only a 43% TF-CBT training completion rate in the first 2 years of their training program. Barnett et al. (2019) reported that the completion rate for participants being trained in a statewide TF-CBT dissemination program was 34%. The authors suspected that some of the drop-outs may be attributed to normal staff turnover. Of course, it should be noted that the lack of a randomly assigned comparison group followed at the same time as the trainees in the present study prohibits concluding that the addition of the PWYP approach increased the completion rate. Nevertheless, given the variability in completion rates across training programs, participant completion rate seems to be an important variable to include in future research comparing the PWYP approach to standard training programs.

The findings demonstrated that clinicians/supervisors reported significant increases in engaging in physical activity, taking deep breaths as a relaxation strategy, writing down thoughts and feelings about a situation, and, more broadly, in personally practicing skills taught to clients. With regard to coping, the clinicians/supervisors also described more frequent use of instrumental social support (speaking to or seeking assistance from others), active coping (taking steps to resolve a problem), humor, and restraint (taking some time to avoid acting too quickly) after their participation in the training program. Consequently, the results suggest that the same skills that clients learn in the context of TF-CBT may be valuable in helping clinicians/supervisors cope with their own personal and professional stressors. Although it is possible that clinicians/supervisors may have described enhanced coping and reduced STS attributable to simply the passage of time or in response to any training that leads them to feel more competent, the current results did find that clinicians and supervisors reported increases in the specific types of coping strategies that the PWYP program had encouraged. Moreover, one of the qualitative statements that was made by a clinician seems to reflect what the present PWYP approach had intended: “I was able to see first-hand the benefits of the skills and feel more confident and passionate about sharing them with my patients.”

The potential benefits of incorporating self-care into training for mental health clinicians/supervisors may have important implications for professional training in a wide array of evidence-based treatment models. Typically, evidence-based treatment models teach skills that have broad scientific support and are applicable to all people including clinicians/supervisors themselves. In general, the findings seem to suggest that encouraging clinicians to “PRACTICE What You Preach” may enhance clinicians’/supervisors’ personal coping and may reduce their levels of STS without compromising their treatment implementation efforts and client outcomes. Incorporating a PWYP approach into training in evidence-based treatment models may lead to enhanced self-care among professionals, potentially reducing the extremely high staff turnover rates endemic at many community mental health settings serving vulnerable populations (Aarons et al. 2011; Swain et al. 2010; Woltmann et al. 2008). With respect to the present study’s limitations, there was no random assignment to a control group for comparative purposes, and there was no long-term follow-up assessment. Consequently, the significant changes that were found might simply be attributed to the passage of time. It was also impossible to control for any differential effects associated with one type of community mental health agency as opposed to another given that 19 different agencies participated in the study.

Future research examining training methods for enhancing the skills of clinicians/supervisors has the potential to identify means of increasing the accessibility of effective treatments for diverse youth impacted by trauma, while potentially enhancing the well-being and stability of community organizations’ mental health staff. A training approach that incorporates a significant emphasis on participant self-care may not only produce treatment fidelity, professional competency, and positive outcomes for children, but may also help to retain therapists in the field longer due to enhanced professional coping as well as reduced STS. Clinicians/supervisors who are less impacted by the stress of this difficult work may be better able to sustain their enthusiasm and effectiveness for addressing the therapeutic needs of the vulnerable population of children and caregivers impacted by trauma.
